# The nature of evidence for and against epigenetic inheritance

**DOI:** 10.1186/s13059-015-0709-y

**Published:** 2015-07-11

**Authors:** Joseph H. Nadeau

**Affiliations:** Pacific Northwest Diabetes Research Institute, 720 Broadway, Seattle, WA 98119 USA

## Abstract

Not so fast. The Iqbal et. al. study and the associated Whitelaw commentary highlight the appropriately high standards of study design and interpretation needed to obtain good evidence for or against epigenetic inheritance.

Please see related article: www.dx.doi.org/10.1186/s13059-015-0714-1

## Correspondence

Recent work in bacteria, plants and animals has provided new evidence for the old idea that phenotypes acquired during a lifetime can be passed to later generations, independently of DNA variation [1]. These revolutionary discoveries suggest that DNA is not the only molecule contributing to phenotypic inheritance. The search is now on to identify the molecular basis for this novel mode of inheritance, establish the rules for segregation and transmission across generations, and characterize the ways that epigenetic changes, often in concert with genetic variation, provide both phenotypic plasticity and evolutionary adaptations. Epigenetics enables developmental and physiological responses to both external (environmental) and internal (genetic) challenges. These adaptations can endure for many generations if conditions persist, but can be reversed or revised if conditions change. Sensitivity of epigenetic features to both environmental conditions and genetic background suggests that study designs require unusual rigor to enable strong interpretations.

Unlike the singular role of DNA in genetic inheritance, many molecules and mechanisms are probably involved in epigenetic inheritance. Epigenetics relies mainly on chemical modifications of nucleic acids and proteins as well as on changes in the numbers, kinds and functions of RNAs. The actions of these molecular modifications are not limited to gene expression, regulatory RNAs, translation, and the function of proteins such as histones and prions can also be affected. Many of these molecular changes occur only in conjunction with specific DNA, RNA and protein sequences. The search space for mechanism is therefore large.

A recent study highlights the challenges of epigenetics research. Iqbal et al. [2] sought to test whether methylation changes show the expected patterns of inheritance, namely that phenotypes and epigenetic features induced in the parental G0 generation are transmitted to the G1, G2 and later generations. For this test, several inbred strains of mice were treated with agents that act as endocrine disruptors (EDs) – molecules that interfere with hormonal control of gene expression. The tested agents, vinclozolin, bisphenol A and a phthalate, were previously shown to induce diverse epigenetically inherited phenotypes such as spermatogenetic abnormalities and infertility [3]. Following parental exposures in the G0 generation, methylation patterns and mRNA levels were altered in the germline of the G1 but not the G2 generations. The authors concluded that ED-induced epigenetic changes were reversed as part of normal processes that reprogram epigenetic features such as imprinting at each generation. In an accompanying commentary, Whitelaw concluded that this study provides important negative evidence concerning epigenetic inheritance [4].

Several issues temper these strong conclusions. Genetic background and treatment protocols are known to influence efficacy of agents that induce epigenetic inheritance [5, 6]. Occurrence of ED-induced phenotypes varies among strains in both rats and mice and with the extent of inbreeding, with reported effects often stronger with outbred versus inbred animals. The Iqbal study did not assess phenotypic changes, apparently assuming that previously reported evidence suffices. In addition, no evidence in the literature or in this report shows that the JF1 strain is responsive to ED-induced phenotypic changes, and in fact the 129 strain was previously reported to be resistant [6]. In addition, different routes of administration (intra-peritoneal injection versus gavage), doses and treatment schedules were used. Such variability in response obligates researchers to demonstrate that the environmental agents induce the expected phenotypes, as is usually done cf. [3, 7, 8]. Absence of direct evidence for ED-induced phenotypes with these strains and protocols compromises clean interpretation of the results of this study.

With respect to molecular features, Iqbal et al. [2] found molecular changes in the G1 generation, but not in the subsequent generation, leading them to conclude that reprogramming reversed these epigenetic marks. But several studies have shown that epigenetic inheritance often involves two independent steps, namely initiation - induction of primary molecular changes, and then propagation - translation to secondary features and their inheritance across generations [9, 10]. In some cases, initial methylation changes are replaced with other modifications in subsequent generations [9]. In other cases, piRNAs in the initial generation trigger histone modifications that modulate phenotypic variation in later generations [10]. It is impossible to know whether the Iqbal study show an initiation but not propagation, or alternatively whether the initial changes were then translated into other epigenetically inherited marks that were not measured. Without phenotypic assessments and epigenetic profiling across generations, these alternatives cannot be resolved.

As Mendel showed in the first test of associations between genotype and phenotype, following the inheritance of phenotypic, genetic and now epigenetic variation across generations is essential. The Iqbal study did not truly test epigenetic inheritance because phenotypes were not characterized. Moreover, failure to find propagated molecular changes does not prove that methylation is not pivotal. To study inheritance, associations between epigenetic, genotypic and phenotypic features must be simultaneously followed. Proving a negative is difficult and the need for rigor is necessarily high. This study and its commentary nicely demonstrates the challenges.

## Abbreviations

ED: Endocrine disruptor
G0, G1, G2: G0 is the treated generation and G1 and G2 are the subsequent generations
